# Privacy considerations for sharing genomics data

**DOI:** 10.17179/excli2021-4002

**Published:** 2021-07-16

**Authors:** Marie Oestreich, Dingfan Chen, Joachim L. Schultze, Mario Fritz, Matthias Becker

**Affiliations:** 1 Systems Medicine, Deutsches Zentrum für Neurodegenerative Erkrankungen (DZNE), Venusberg-Campus 1/99, 53127 Bonn, Germany; 2 CISPA Helmholtz Center for Information Security, Saarbrücken, Germany, Stuhlsatzenhaus 5, 66123 Saarbrücken, Germany; 3 Genomics and Immunoregulation, Life & Medical Sciences (LIMES) Institute, University of Bonn, Bonn, Germany, Carl-Troll-Straße 31, 53115 Bonn, Germany; 4 PRECISE Platform for Single Cell Genomics and Epigenomics at Deutsches Zentrum für Neurodegenerative Erkrankungen (DZNE) and the University of Bonn, Germany, Venusberg-Campus 1/99, 53127 Bonn, Germany

**Keywords:** data privacy, data sharing, genomic data, transcriptomic data, epigenomic data

## Abstract

An increasing amount of attention has been geared towards understanding the privacy risks that arise from sharing genomic data of human origin. Most of these efforts have focused on issues in the context of genomic sequence data, but the popularity of techniques for collecting other types of genome-related data has prompted researchers to investigate privacy concerns in a broader genomic context. In this review, we give an overview of different types of genome-associated data, their individual ways of revealing sensitive information, the motivation to share them as well as established and upcoming methods to minimize information leakage. We further discuss the concise threats that are being posed, who is at risk, and how the risk level compares to potential benefits, all while addressing the topic in the context of modern technology, methodology, and information sharing culture. Additionally, we will discuss the current legal situation regarding the sharing of genomic data in a selection of countries, evaluating the scope of their applicability as well as their limitations. We will finalize this review by evaluating the development that is required in the scientific field in the near future in order to improve and develop privacy-preserving data sharing techniques for the genomic context.

## Introduction

Since the introduction of high throughput data generation methods, the medical field has become increasingly data-driven. Large biomedical studies often comprise sampling and data generation at different centers, requiring the data to be subsequently shared for joint analysis. Though data sharing has thus become crucial, the inherently private nature of medical data demands caution and elaborate sharing protocols are necessary to ensure patient privacy. This is particularly important when sharing genomics data and the associated medical metadata. The concise risks of privacy breaches and the methods used by adversaries vary for different data types. In this review, we will assess such risks and techniques in the context of genomic data, more precisely, three different data types: genomic sequences, transcriptomic and epigenomic data. We will further elaborate on currently used methods for privately sharing data along with different laws that are meant to protect individuals in case of a data leak. We will additionally introduce current research areas for private sharing of genomic data and give an outlook on the necessary as well as expected changes in the upcoming years.

## Genomic Data Types

**Figure 1:**
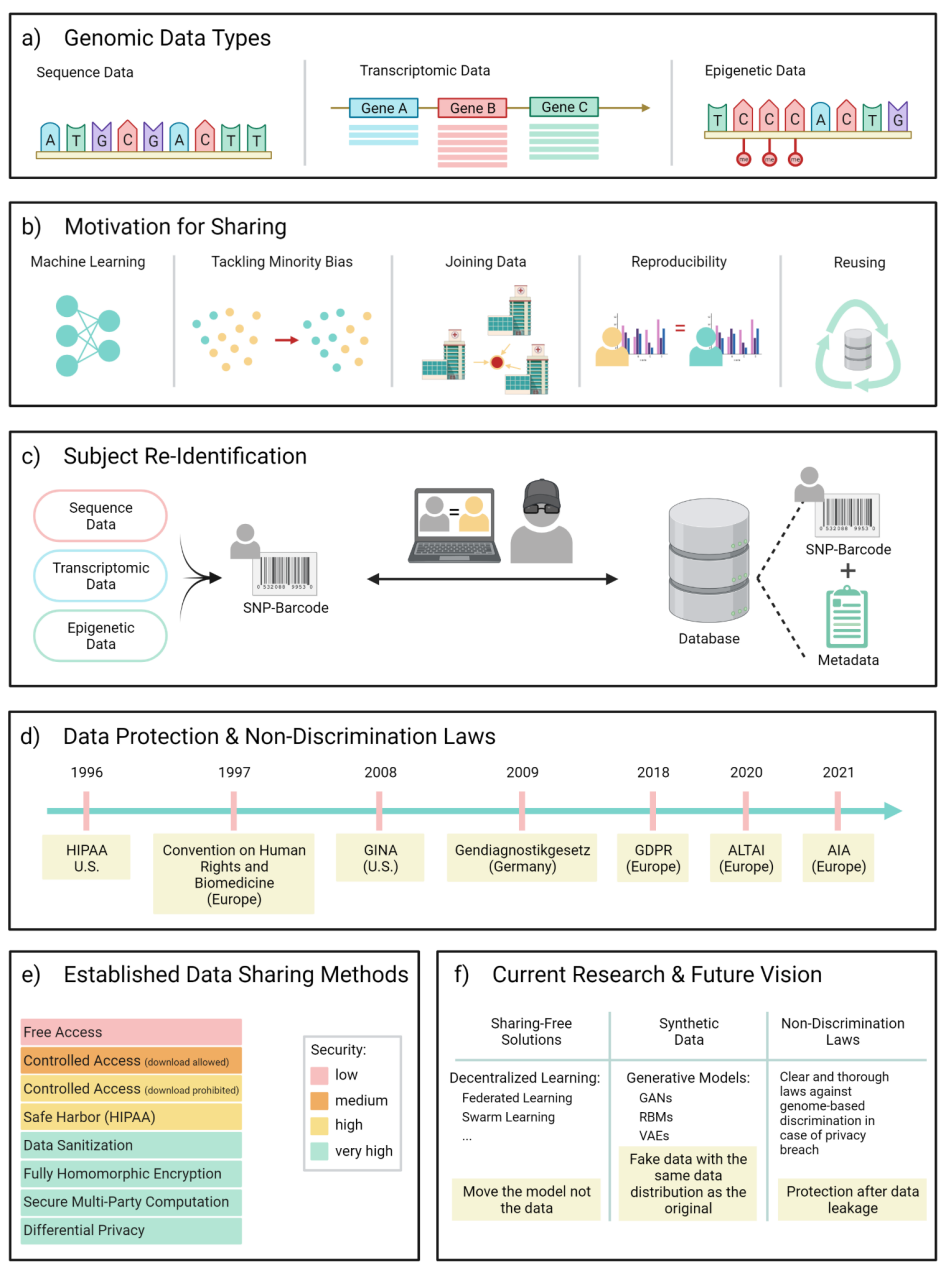
Brief overview over the contents of this review. a: The three different types of genomic data that are covered in this work. A: Adenine; C: Cytosine; G: Guanine; T: Thymine; me: methyl group. b: Shown are a selection of applications that encourage data sharing. From left to right: Genomic data sharing is often required when building machine learning models in order to increase the available sample size required for training. Collecting and enriching data on minorities can reduce subpopulation bias in a trained model. Data often needs joining in multiparty studies when it is collected at different sites. Other motivators are sharing genomic data to allow the reproducibility of results or to reuse the data for new scientific questions. c: The subject re-identification is the core concern in genomic data privacy. The ability to produce uniquely identifying Single-Nucleotide-Polymorphism(SNP)-barcodes from the data allows an adversary to cross-reference these with public databases, often containing meta information that give away sensitive medical information. d: A timeline of selected laws that were introduced in several countries to protect citizens from discrimination based on genome-related data. e: Displayed are a selection of commonly used data sharing methods, colour-coded based on the maximum level of security they can provide. f: Selection of upcoming sharing techniques that are subject of ongoing research. Also shown as a necessary future step is the invocation of globally valid laws to protect subjects from discrimination in the case of a security breach. GAN: Generative Adversarial Network; RBM: Restricted Boltzmann Machine; VAE: Variational Autoencoder.

Traditionally, genomic data (Goodwin et al., 2016[36]) refers to data holding information on the base sequence in an individual's genome. Here, we are going to extend this notion of genomics data to also incorporate transcriptomic as well as epigenomic data, which are closely associated with and influenced by the genomic sequence (Figure 1a(Fig. 1)). This serves as a more holistic assessment of potential privacy breaches when working with either category of data by evaluating how these different data types impact and relate to one another and how weak points may translate across categories.

### Sequence data

Sequence data contains qualitative information, identifying specific bases and their positions along the genome. This data type describes an individual's genomic sequence to a different extent, depending on the protocol underlying the data generation process. Whole genome sequencing (WGS) produces genomic sequences in their entirety, while partial genomic sequencing focuses only on particular areas, e.g., exon regions in case of whole exome sequencing (WES) (Maróti et al., 2018[53]). Even more condensed is sequence data storing only single nucleotide polymorphisms (SNPs) (Robert and Pelletier, 2018[63]), which are specific sites in the genome for which different allele frequencies have been observed between populations. Despite its strongly reduced dimensionality, SNP data often allows for accurate genomic fingerprinting of individuals (Lin et al., 2004[50]).

### Transcriptomic data

Unlike sequence data, transcriptomic data is of a quantitative nature. It stores information on the abundance of RNA transcripts in a sample, mostly gene transcripts in the form of mRNA, but also other types such as long non-coding RNAs or microRNAs. Transcript abundances are directly influenced by external factors such as environmental stressors, pathogens, or drugs but are also closely impacted by the underlying genomic sequences. Genomic mutations can change a gene's susceptibility for transcription and are therefore indirectly reflected in the transcriptomic landscape. The genomic locations that are associated with variation in transcript abundances are referred to as expression quantitative trait loci (eQTLs) (Nica and Dermitzakis, 2013[56]). Transcriptomic data can be collected at different levels of resolution: more coarse grained data is measured using bulk-RNA-sequencing, where transcript abundances are measured across many cells, which is in contrast to the higher resolution of single-cell RNA-sequencing, where transcripts are quantified per cell (Tang et al., 2009[70]). 

### Epigenomic data

Epigenomic data holds information on heritable alterations that affect gene expression but are not based on changes in the genomic sequence itself (Weinhold, 2006[77]). These alterations typically take place on the DNA or on histone proteins that are responsible for binding and condensing the DNA, therefore impacting its accessibility for transcription. One important modification on the DNA itself is DNA methylation, which commonly occurs on cytosine bases that are followed by a guanine, so called CpG dinucleo-tides. These often occur in dense clusters referred to as CpG islands. In general, DNA methylation is associated with a reduction of gene expression whereas the absence of methylation promotes RNA transcription. Histone modifications are post-translational alterations on histone proteins that affect the histone's ability to remodel the chromatin. They include - among others - methylation and acetylation on amino acid rests and impact how densely the histone can adhere to the DNA (Cazaly et al., 2019[11]). There are other levels of epigenomics data that are determined by sequencing, for example, so-called open chromatin regions, regions that can be accessed by transcription factors to regulate gene transcription. Out of these types of epigenetic data, in this review, we will focus on DNA methylation data, given that it may be directly impacted by changes in the genomic sequence.

## Why Share Genomic Data?

In order to assess privacy issues that arise when sharing genomic data, there is a need to discuss the motivation behind sharing it in the first place (Figure 1b(Fig. 1)). 

Data sharing is required during multi-party studies, where separate datasets have been collected in a decentralized fashion and subsequently have to be joined together. Further, sharing is required to improve the reproducibility of results by publicly (or on request) providing the dataset that was used in a particular analysis. A set of principles targeting the findability, accessibility, interoperability and reusability of data, particularly by machines, was introduced by Wilkinson et al. as the FAIR-principles (Wilkinson et al., 2016[78]). Other scenarios that require data sharing are the reusing of data, in which case datasets are shared for further use in order to answer questions other than those originally posed when collecting the data, or sharing for reduction of model bias. The latter is especially important when the data was collected from minority groups, in which case public accumulation can battle common bias problems in population-based modeling approaches (Mehrabi et al., 2019[54]).

A rather recent development that urges the distribution of genomic data is the increasing application of machine learning techniques (Libbrecht and Noble, 2015[49]). These approaches typically require a large amount of training data, especially in the context of deep learning (Eraslan et al., 2019[24]), as otherwise the model tends to bias towards local minima that generalize poorly on unseen samples. The demand for big data is particularly strong in genomics because of its immense feature space, and oftentimes exceeds the scope of a single study by far, prompting the collection of data from multiple studies. 

Moreover, researchers (Brittain et al., 2017[10]; Dand et al., 2019[18]) and politicians (The White House, 2015[71]; BMBF, 2018[8]) alike are promoting the advent of personalized medicine to enable the development of treatment plans tailored to the individual. To provide the knowledge base for personalized medicine, a great wealth of biomedical data, including genomics data, will have to be generated and shared, given that the sampling will have to be conducted in a decentralized fashion to properly reflect human diversity and the data has to be accumulated for subsequent analysis. Given the highly private nature of the data, this naturally demands a thorough discussion on how to guarantee sufficient privacy compliance.

## Privacy Concerns when Sharing Data

In light of the aforementioned reasons to share genomic data and before introducing concrete data sharing methods, the problems that arise from privacy breaches in the context of genomics must be elucidated. The root of these problems and the reason why genomic data privacy requires thorough discussion is the issue of subject re-identification. Identifying the individual which the data was taken from can potentially have severe consequences starting with social issues such as stigmatization, which is often observed when there is knowledge about the presence of certain risk alleles in an individual's genome, especially in the context of mental health issues (Ward et al., 2019[75]). Additionally, known preconditions and increased disease risks can negatively affect chances for employment or health insurance (Godard et al., 2003[34]). There have also been cases where adopted children or children received by use of sperm donations identified their biological parent(s) due to publicly available genomic information (Erlich and Narayanan, 2014[26]). Another issue is posed by the longevity of genomic data: given the heritability of genomic and epigenomic alterations, re-identification does not only pose a risk to the individual itself but also to close relatives (Ayday et al., 2015[3]; Oprisanu et al., 2019[58]). While all living relatives can - in theory - be asked to give their consent to the collection of the data in addition to the consent given by the individual, privacy breaches may also impact unborn relatives or underage relatives whose consent cannot be given. 

The risk of re-identification and the corresponding instantiated attack methods are different for the previously introduced data types. We will first discuss these with respect to sequence data and we will see that most re-identification risks associated with transcriptomic and epigenomic data fall back onto techniques that infer the underlying sequence information. 

### Sequence data

One particular privacy complication often discussed in the context of sequence data is SNP “barcoding” or “fingerprinting”. This refers to the observation that a limited number of SNPs - 30 to 80 independent SNPs according to (Lin et al., 2004[50]) - is sufficient to unequivocally identify an individual, therefore providing a genome-based fingerprint of that person. Following this concept, an attacker can construct a SNP fingerprint-library from all publicly available sequence datasets and identify individuals - given their DNA - by checking for matches with fingerprints of publicly deposited sequences, which are often associated with study metadata that reveals some of the individual's medical information (Figure 1c(Fig. 1)). This scenario raises the question of how an attacker would obtain the victim's DNA sequence in the first place. Some examples here are DNA theft or careless publication of sequence data. The former is often illustrated by means of the coffee-cup example, where a malicious attacker acquires a person's DNA by collecting it from a thrown-away coffee cup (Gürsoy et al., 2020[37]). There are of course many other ways to steal DNA, be it from a tossed-out tissue, blood, or other sources as long as they contain intact cells. To then go from the sample to the DNA sequence is easier than oftentimes assumed, nowadays, where companies have specialized in direct-to-consumer individual genome sequencing for a moderate price, often only requiring as much as a saliva sample (Eissenberg, 2017[22]). The privacy concerns that accompany SNP fingerprinting translate further onto other types of genomic data, if the data allows the inference of the underlying SNPs.

A prominent example of the careless publication of private sequencing data occurred during the Personal Genome Project. Here, a group from Harvard College demonstrated that many of the uploaded, publicly available files contain personal information, such as the individual's first and last name that was part of the file identifiers after decompression (Sweeney et al., 2013[69]). 

But re-identification using sequence data does not always have to be part of a malicious attack. A rather recent example is the apprehension of the “Golden State Killer”, who was caught several decades after his first known crimes due to the use of consumer genetic databases. Using the criminal's sequence data and the data available in those databases, the investigators reconstructed several family trees by pairing the database information with public records, ultimately leading to the apprehension through distant relatives who had uploaded their genomic sequences (Zabel, 2019[82]). Though this case of re-identification helped to catch a serial killer, it also explicitly emphasizes privacy concerns in the context of shared genomic sequences.

There have been cases of deliberate sequence data publication in which particularly sensitive parts of the genome were removed to prevent the individual's risk assessment with respect to certain diseases. A well-known example is the publication of James Watson's whole genome with the exception of the APOE-gene. The gene has been associated with late onset Alzheimer's disease - information that was not meant to be revealed. Shortly thereafter, Nyholt et al. (2009[57]) demonstrated that the removal of the gene alone does not necessarily prevent the accurate prediction of the disease risk posed by different alleles of the gene, since these were shown to be in linkage disequilibrium with other SNPs which had not been removed.

In cases where publicly available sequence data is available in combination with corresponding meta information, an individual's sequence does not necessarily have to be acquired to re-identify the individuals in the dataset. This is due to the personal information contained in the meta information. The risk posed by metadata has been significantly mitigated by means of legal regulations, however, free-form text, longitudinal data, low sample size (El Emam, 2011[23]), and non-random generation of accession numbers (Erlich and Narayanan, 2014[26]) have been shown to remain problematic. 

A thorough review of the requirements for secure genomic sequence sharing, storing, and testing methods is provided by Ayday et al. (2015[3]).

### Transcriptomic data

Already in 2012, Schadt et al. demonstrated how to infer an individual's genotype at eQTL positions and therefore to SNP-fingerprint the individual using a Bayesian approach that solely relied on publicly available data on putative *cis*-eQTLs and RNA expression data (Schadt et al., 2012[65]). They pointed out that this is particularly problematic given that gene expression data is commonly assumed to allow too little insight into a study participant's genomic sequence to reveal their identity and has therefore been shared publicly on platforms such as ArrayExpress and Gene Expression Omnibus, while sequence data is held under controlled access. However, if contrary to common belief, gene expression data does allow SNP-fingerprinting, then the publicly available data for building a SNP-fingerprint library for re-identification attacks mentioned in the sequence section above is expanded from publicly accessible sequence data to both sequence data and gene expression data, resulting in a much larger pool of individuals at risk of re-identification. The paper was reviewed by Erlich and Narayanan (2014[26]) who concluded that the threat posed to individuals whose gene expression data has been published is low. This conclusion was based on the fact that the Bayesian method introduced by Schadt et al. only performed well when the eQTL data and gene expression data were measured on the same platform, while at the time both sequencing on microarray and Next Generation Sequencing (NGS) were common, making many of the datasets incompatible for the inference approach. In 2017, however, Lowe et al. (2017[52]) showed that the use of RNA-seq surpassed that of microarrays in 2016, showing increasing trends for RNA-seq while microarray publications decreased further in count. Corchete et al. (2020[15]) referred to RNA-seq as the “first choice in transcriptomic analysis” in 2020. Thus, while being reasonable at the time, the argument that platform heterogeneity hinders phenotype inference in many cases progressively loses its validity when the sequence data landscape becomes increasingly homogeneous. Erlich and Narayanan further argued that the amount of public gene expression data to be downloaded and processed by an adversary to generate the SNP barcodes requires immense computational power rendering it an unlikely endeavor. Putting this into today's perspective, with the currently available hardware, new compute architectures (Becker et al., 2020[5]), and cloud space for rent, this argument as well requires reassessment. This emphasizes the need for re-evaluation of the re-identification risk posed by the method proposed by Schadt et al. considering the technological changes that have occurred since its original publication. The computational feasibility of SNP-fingerprinting entire databases such as GEO would enable an attacker to test whether a given individual - e.g., crime suspect, victim of DNA theft, person with public genome sequence - is part of a study in which the gene expression data has been published. Gene expression data could then also be utilized for associating individuals with certain traits such as BMI, sex, age, insulin levels and glucose levels (Schadt et al., 2012[65]) or to match individuals of one study with those of another, creating cross-links that potentially reveal further meta information on an individual. These genotype-phenotype linking attacks have been assessed in more detail by (Harmanci and Gerstein, 2016[40]), urging to use methods to quantify private information leakage before publishing a dataset.

### Epigenomic data

The methylation of DNA is measured using bisulfite conversion. Here, DNA is treated with sodium bisulfite which does not impact methylated Cytosines (C) but converts unmethylated ones into Uracil (U). Adenine (A), Guanine (G) and Thymine (T) are not affected. C/T-SNPs, i.e. SNPs where a C was changed to a T, are the most common transitions in human genomes (LaBarre et al., 2019[47]) and they can change a CpG dinucleotide into a TpG dinucleotide. These SNPs often induce a three-tier pattern in the measured methylation according to homozygous TpG individuals with low, heterozygous CpG/TpG individuals with medium and homozygous CpG individuals with high methylation signals. Similar effects could be observed with C/A or C/G mutations (Philibert et al., 2014[60]; Daca-Roszak et al., 2015[17]). Based on these tri-modal patterns, LaBarre et al. developed a method that recognizes C/T-SNPs in methylation data and subsequently removes them. However, the three-tier patterns could also result from differential methylation rather than underlying genotypes, and SNPs in CpGs that are always unmethylated are unlikely to be detected, since it is not possible to distinguish between a C/T-SNP and a C that is converted to a T during bisulfite conversion. 

In 2019, Hagestedt et al. showcased an effective membership inference attack on DNA methylation data, i.e., inferring the presence of an individual in a genomic dataset, disclosing the privacy risks posed by their public release (Hagestedt et al., 2019[38]). They further introduced MBeacon, a platform where researchers can query DNA methylation data deposited at Beacon sites with regard to whether or not they contain samples with specific methylation. MBeacon was designed using a privacy-by-design approach, substantially decreasing the success of membership inference attacks while maintaining a decent level of data utility.

Besides the potential inference of the underlying genotype, another privacy concern was raised by Philibert et al. (2014[60]). They claim that the methylation status of an individual at specific sites could be used to infer the smoking status and alcohol consumption of the individual. A response was issued in 2015 by Joly et al. (2015[44]), members of the International Human Epigenetic Consortium (IHEC), criticizing that the prediction of smoking status was not accurate enough to give reliable results and that the risk of re-identification is low in the absence of access to the corresponding genomic sequence information. Dyke, together with other authors that also participated in the response letter of Joly et al., issued a study that year which illustrates the inference of SNPs from DNA-methylation data using imbalances in methylation signal from forward and reverse strands when the data was collected using strand-specific Whole Genome Bisulfite Sequencing (WGBS) (Dyke et al., 2015[21]). They emphasize that the risk of re-identification is low but may be increased in combination with meta information on the individual's demographic or health status, especially in the case of rare disease phenotypes. To maximize the privacy of participants in DNA-methylation studies, the authors ask for a more synchronized metadata vocabulary, in order to avoid different levels of information being revealed by different authors based on the descriptors they used. They also recommend that CpGs overlapping known SNPs are removed from DNA-methylation data prior to publication and offer a list of questions to consider to protect people with rare diseases in particular.

Berrang et al. provided a privacy risk assessment spanning different types of genomic data along a temporal axis and between related individuals (Berrang et al., 2018[6]). They demonstrated a Bayesian framework that utilizes correlations present between different data types to infer the values of one data type, using the information from another, e.g., inferring the methylation pattern from sequence data and vice versa. 

## Laws and Limitations

Many countries have developed sophisticated laws and guidelines to protect individuals from misuse of their genomic information (Figure 1d(Fig. 1)). However, the wording is often ambiguous towards the different types of genomic data and has therefore prompted researchers to question their validity in particular situations. 

For example, the German “Gendiagnostikgesetz” prohibits discrimination of citizens based on their genetic characteristics. Genetic characteristics are here defined as inherited or between conception and birth acquired hereditary information of human origin (Deutscher Bundestag, 2009[19]). This unambiguously covers genomic sequence information as it is present at birth. It is unclear, however, if this also covers quantitative gene expression information which can be affected by the environment and not only by underlying sequence information. Further, if it covers epigenomic data, which is often altered by environmental factors and therefore can be lost or acquired after birth and is not necessarily inherited. Lastly, if it also refers to genomic sequence information that has changed after birth, e.g., mutations due to external stimuli such as UV-radiation. The law also states that it does not apply to genetic examination and analyses and the handling of genetic samples and data for the purpose of research. The Council of Europe had previously issued a convention in 1997 that prohibits “any form of discrimination against a person on grounds of his or her genetic heritage”, where the term genetic heritage is not defined further. This convention was signed and ratified by 29 countries (Council of Europe, 1997[16]). In the United States, a similar effort has been made to protect citizens from discrimination in health insurance and employment based on genetic information by introducing the Genetic Information Nondiscrimination Act (GINA) (U.S. Equal Employment Opportunity Commission, 2008[73]). Genetic information is here defined as information on an individual's genetic test, genetic tests of their family members and diseases or disorders that have manifested in family members of the individual. A genetic test is defined as “an analysis of human DNA, RNA, chromosomes, proteins or metabolites, that detects genotypes, mutations, or chromosomal changes”. This definition leaves the same questions unanswered as above. Additionally, it explicitly excludes the U.S. military from the list of employers that are prohibited to use genetic information as well as any employer with less than 15 employees. 

In addition to GINA, disclosure of health information is regularized by the Health Insurance Portability and Accountability Act (HIPAA). It provides three standards for the disclosure of patient health data that do not require authorization by the patient. Those three standards are the Safe Harbor standard, the Limited Dataset standard, and the statistical standard (El Emam, 2011[23]). Safe Harbor regulates the de-identification of health data by removing 18 different identifying elements, among those the name, certain geographic information, all elements of dates except for the year, phone and fax numbers, e-mail addresses, social security numbers, and many more (El Emam, 2011[23]). The Limited Dataset standard only removes 16 potential identifiers but additionally requests a data sharing agreement between data custodian and data recipient and the statistical standard requires expert evaluation and classification of the de-identification risk as very small (El Emam, 2011[23]). Given its clarity and simplicity, Safe Harbor is often referred to for data de-identification. However, as pointed out by El Emam, not only does it often result in the removal of information that could have been useful for the data evaluation, it also does not sufficiently ensure the protection of the individual with regard to re-identification. In this context, there is special emphasis on the lack of protection through genetic data, such as SNPs, longitudinal data, widely used diagnosis codes, sampling size, and free-form text. A more detailed elaboration can be found in El Emam (2011[23]).

In 2018, major advancements have been made in the EU with respect to data protection due to the release of the General Data Protection Regulation, GDPR (European Union, 2018[30]; Shabani and Borry, 2018[66]). It applies in all EU member states and aims to unify data protection across countries. GDPR overcomes the ambiguous definition of genetic data as outlined above by defining it as “personal data relating to the inherited or acquired genetic characteristics of a natural person which give unique information about the physiology or the health of that natural person and which result, in particular, from an analysis of a biological sample from the natural person in question” (“Recital 34 - Genetic data - GDPR.eu,” 2018[33]). This definition includes the wide range of modern genomics data types and therefore offers more thorough protection from open sharing and un-consented processing than the regulations discussed above. While passed in the EU, the law applies worldwide if the data that is processed was sampled from a citizen of an EU member state.

Additional EU guidelines have been introduced recently that specifically aim to protect EU citizens from threats posed by Artificial Intelligence (AI) systems. The “Assessment List for Trustworthy Artificial Intelligence” (ALTAI) was published by the European Commission in 2020 as a guideline for the development of trustworthy AI (European Commission, 2020[28]). Early in 2021, the European Commission additionally published a draft of the Artificial Intelligence Act (AIA) (European Commission, 2021[29]). Subject to this act is any provider of AI applications worldwide, if those applications are used by EU citizens. It categorizes AI systems into different categories of threat, connected with strict obligations that have to be met by the provider. These regulations also address AI used in the health context, therefore including genomic data. While the motivation behind these guidelines and regulations is most reasonable, there are some concerns regarding the implications they might have on data privacy. This is particularly important because they demand the models to be fair and unbiased by using representative, non-discriminatory and complete training, validation and test datasets. However, in order to test a model's fulfillment of these requirements, the inspecting authority is likely to need access to the datasets, which - e.g., in the context of genomic data - are often highly private.

## Risk-Benefit Considerations

When assessing privacy risks, it is always essential to weigh the risk of an individual against the possible gain that is associated with the vulnerable position the individual finds themself in. The group of individuals that was - and in many scenarios still is - the main target groups for genomic data collection are those that have a personal reason to participate in the respective studies, e.g., a difficult-to-treat or poorly researched disease (Esplin et al., 2014[27]). For these individuals, the potential gain that comes from participating in these studies often substantially outweighs the risk of re-identification. But the focus of the target group appears to gradually shift, people have started to have their genome sequenced out of curiosity rather than acute medical reasons and the field of personalized medicine advocates genomic data collection to become part of a medical care routine (Brittain et al., 2017[10]; Suwinski et al., 2019[68]). Therefore, the group at risk of re-identification is bound to change from those that have a high benefit-risk ratio to a more heterogeneous population. Additionally, the risk of cross-referencing genomic data and metadata to narrow down the set of individuals a data instance potentially belongs to, is likely to increase due to oversharing of personal information on social media, be it voluntary or involuntary. This can start with information as subtle as height, sex, and weight which can be inferred from pictures, geotags, and dates that put an individual into close spatial and temporal proximity of the conduction of a given study, and it can go as far as people openly sharing their health status or study participation. This can be expected to substantially increase the risk of re-identification and it is an issue that has lacked thorough attention in prior risk-evaluation strategies.

## Established Data Sharing Techniques

Current data sharing techniques come at different levels of security, as is illustrated in Figure 1e(Fig. 1). As discussed above, some data types are often shared publicly in plain text without restrictions other than de-identified sample descriptors. This is the case when the risk of re-identification for the participants is considered minimal. Genomic data with increased re-identification risk such as sequence data or data that gives direct information on partial sequences (reads, SNPs) are published under controlled access. In this scenario, the legitimacy of an access request is evaluated based on the applicant's personal information and the research project the data is intended to be used for. The use of the data also often comes with a series of constraints that regard a safe storing location, no sharing and no re-identification attempts. While there is still a lack of true oversight with respect to whether or not the data is shared after downloading, another controlled access strategy is to not allow the data to be downloaded but instead run protocolled queries on the data and only retrieve the results. This may severely restrict the flexibility with which the data can be analyzed (Erlich and Narayanan, 2014[26]). 

To decrease the risk of subject identification, be it in publicly shared or controlled-access data, efforts are made to de-identify the data, i.e., to remove information or reduce its granularity such that identification of the individual becomes very unlikely. A common set of guidelines for de-identification is the mentioned Safe Harbor standard included in the HIPAA Privacy Rule. 

Gürsoy et al. developed a method that sanitizes the raw reads underlying gene expression data such that sharing with reduced re-identification risk is possible, while keeping the necessary data manipulation minimal (Gürsoy et al., 2020[37]). They achieve this by transforming the original BAM file into a sanitized file, where information that reveals the presence of a variant (SNPs, insertions, deletions) is masked. For instance, information on variants as it is contained in reads is removed by replacing the called base at the site of the variant with that present in the reference genome. The true values of the sanitized elements are stored in a separate file which is meant to be under controlled access. This allows for sharing of the sanitized data while being able to reconstruct the original if access to the additional file is granted, though no formal or statistical guarantees on privacy are provided.

Classical encryption approaches have been leveraged as well to enable secure sharing of genomics data. One example is the crypt4gh file format introduced in 2019 by the Global Alliance for Genomics and Health (GA4GH) (“GA4GH File Encryption Standard,” 2019[32]). The format allows the genomic data to be encrypted while in storage, in transit, during reading and writing. It uses symmetric encryption as well as public-key encryption, it is confidential in the sense that it is only readable by holders of a secret decryption key, but it does not obscure the length of the file. The data is stored in blocks, the integrity of which is ensured using message authentication codes, however, blocks can be rearranged, removed, or added. Authentication of files encrypted with this method is not provided. Though as with any system that relies on secret decryption keys, privacy is lost in the case of a system breach that results in an untrusted party acquiring the key. 

Others have explored fully homomorphic encryption (FHE) to securely operate on genomic data. FHE allows to conduct computations on the genomic data while it remains in its encrypted state, receiving the encrypted results and decrypting them with a personal key (Erlich and Narayanan, 2014[26]). This allows for secure computations on cloud systems even if the system itself is not. While this approach was long assumed too computationally expensive to be reasonable in the context of genomic data, Blatt et al. recently presented an improvement in run-time of FHE for Genome Wide Association Studies (GWAS) by introducing parallelization and crypto-engineering optimizations, which allegedly outperforms secure multiparty computations (SMPC) (Blatt et al., 2020[7]). SMPC is another approach to secure computation, in which two or more parties that hold private data can compare and perform computations on the data without ever revealing the data itself to the other party or another third party (Erlich and Narayanan, 2014[26]). While a prominent point of criticism with SMPC models is the oftentimes extensive communication required between computing parties, Cho et al. demonstrated an approach for SMPC in GWAS in which run-time scaled linearly to the number of samples and was reduced to 80 days for 1 million individuals and 500,000 SNPs (Cho et al., 2018[14]). Though this is still a substantial amount of time, efforts such as this have worked on optimizing the procedure in the past years. However, in the case of Cho et al., the privacy guarantee only holds for the semi-honest security model, in which participants are assumed to not deviate from the conduction protocol. 

Besides homomorphic encryption and multi-party computing, there are also hardware-based approaches to handling sensitive information such as genomic data. An example is the Intel Software Guard Extension (SGX), which has also been used in combination with homomorphic encryption on GWAS data (Sadat et al., 2019[64]). While such techniques based on SGX can also be used to protect model and/or data (Hanzlik et al., 2021[39]), implementations of SGX however have been troubled with serious security issues themselves (Van Bulck et al., 2018[74]; Lipp et al., 2021[51]).

Another widely applied concept to allow privacy preserving data analysis is that of Differential Privacy (DP) (Dwork and Roth, 2014[20]). The idea behind DP is to allow private data analysis by assuring that the addition or removal of a subject to or from the dataset does not significantly alter potential query results and therefore does not disclose whether or not the individual is part of the dataset. To achieve this, different levels of noise have to be added to the data before its release, where the amount of noise necessary increases when the sample size of a dataset decreases. The privacy loss of the noised dataset is quantified using the epsilon parameter, where a value of 0 indicates total privacy, though decreasing values come with increasing added noise and therefore less utility. In contrast to security measures, cryptography or privacy heuristics, DP comes with strong guarantees that are not susceptible to misuse of the system or typical breaches. Although privacy preserving and statistical analyses do not follow an adverse goal, today's techniques are often affected by a reduced utility of the analysis. For an exhaustive review of privacy-enhancing technologies in the genomic context, please refer to the work done by Mittos et al. (2019[55]).

## Current Research and Future Vision

### Sharing-free solutions

In the machine learning domain, increasing interest has been focused on developing solutions that do not require data to be shared but that store the data locally and have the model migrate instead, performing local training and only sharing updated model parameters (Figure 1f(Fig. 1)). The most prominent sharing-free learning framework is federated learning (FL), whose advantages and major open problems have been discussed thoroughly in recent works (Kairouz et al., 2019[45]; Rieke et al., 2020[62]). FL is a general learning paradigm that can be built on top of various training algorithms, with no restriction on the adopted model architecture, therefore offering a broad application spectrum spanning across diverse data modalities. There have been isolated use cases in the health sector, such as FL for predictions based on electronic health records (Brisimi et al., 2018[9]; Xu et al., 2021[80]), medical images (Sheller et al., 2019[67]; Li et al., 2020[48]) and compound-protein-binding data (Rieke et al., 2020[62]). However, a broad application has so far been hindered by technical obstacles such as the requirement for a somewhat standardized data format that the different sites adhere to, different hard- and software environments, and most importantly, related privacy issues. While FL advertises inherent data security by leaving the data in place, potential information leakages could originate in the transmission of model parameter updates and threats are posed by the untrusted central server that receives and combines the local model updates reported by each site. For example, untrusted servers are shown vulnerable to attacks that reconstruct raw user data from the parameter updates sent by local clients (Zhu and Han, 2020[83]). To minimize the potential privacy risk posed by a vulnerable centralized server, efforts have been made to explore fully decentralized (peer-to-peer) topologies where no central server is required. One completely new data-sharing free approach combining peer-to-peer functionality using blockchain technology with AI is swarm learning (SL) (Warnat-Herresthal et al., 2021[76]). In particular, SL addresses the issue of untrusted participants by registering and authorizing all participating sites via blockchain technology in a swarm network.

### Synthetic data

Another ongoing attempt to boost patient privacy when sharing genomics data is the use of synthetic data instead of the original data. The idea behind this is to create a synthetic cohort that follows a similar distribution as the original cohort, producing accurate analytical results while protecting patient privacy by generating novel samples distinct from the original data (Figure 1f(Fig. 1)). In this regard, researchers have utilized the concept of generative modeling, applying for example Restricted Boltzmann Machines (RBMs) (Hinton et al., 2006[43]) or Generative Adversarial Networks (GANs) (Goodfellow et al., 2014[35]; Yelmen et al., 2021[81]). Though not with the goal to create entire synthetic datasets, variational autoencoders (VAEs) (Kingma and Welling, 2013[46]) have been used to impute missing counts in single-cell expression data (Eraslan et al., 2019[25]; Qiu et al., 2020[61]) as well as in bulk-RNA sequencing and methylome data (Qiu et al., 2020[61]). However, this research is still in its infancy and requires thorough assessment with respect to both the utility and the privacy properties of the generated data. Conventionally, generative models are evaluated along three fronts: (1) fidelity - whether the generated samples can faithfully represent real data; (2) diversity - whether the generated data is diverse enough to cover the variability of real data; and (3) generalization - whether the generated samples are merely copies of real data, i.e., the model overfits and memorizes training data (Alaa et al., 2021[2]). The privacy property started to be considered and investigated in recent works, mostly in the form of membership inference attacks. Specifically, Hayes et al. introduce membership inference attacks against GANs trained on image data (Hayes et al., 2019[41]; Hilprecht et al., 2019[42]) and a systematic analysis has been conducted by Chen et al. (2020[13]). They assess an attacker's ability to infer the presence of a given sample in the GAN's training set with respect to different threat models, dataset sizes, and GAN model architectures. They show that membership inference is facilitated if the training dataset is small, which they explain by the GAN's inability to generalize and instead memorize. This could be an especially limiting factor in the generation of synthetic genomics data since the real data available for training is often limited, especially in the human context, emphasizing the need for privacy risk assessment prior to the public release of the synthetic data or trained models. 

Recently, Oprisanu et al. extended the investigation of GANs' privacy properties to genomic sequence data (Oprisanu et al., 2021[59]). Generally, more research is necessary to improve the utility of generative models while avoiding information leakage and to deploy them to genomics data other than sequences. 

Other work has focussed on private deep learning in general (Abadi et al., 2016[1]) and GANs in particular (Xie et al., 2018[79]; Beaulieu-Jones et al., 2019[4]; Frigerio et al., 2019[31]; Torkzadehmahani et al., 2019[72]) over the past years, among those a recent publication that addresses privacy issues when sharing sensitive data or when sharing generators trained on such data and proposes the training and release of differentially private generators instead (Chen et al., 2020[12]). They further illustrate how the approach can naturally adapt to a federated learning setting. The differential privacy is achieved by restraining the impact a single sample can have on the fully trained model by using differentially private stochastic gradient descent during the training procedure. They further tackle the utility-privacy tradeoff by only training the generator - which may subsequently be publicly released - in a differentially private manner while training the discriminator optimally, therefore not differentially private and discarding it afterwards. Additionally, they demonstrate the value of the approach in a decentralized learning context such as in federated learning approaches. One of the main benefits of a differentially private generator and the obtained samples is that such synthetic data can be accessed without further privacy costs, while, in contrast, differentially private analysis of data needs to keep track of the incurred privacy cost. Also, established tools can be used on such synthetic data, while conventional differential private analysis typically needs to adapt the whole toolchain.

### Protecting privacy and preventing discrimination

Beyond these research-based approaches, more appropriate laws are required worldwide. There needs to be active exchange between the scientific community - particularly medical and life science researchers as well as scientists from the fields of data privacy and data security - and the lawmakers to ensure that the regulations are up to date with the scientific progress and that their phrasing is less ambiguous. The terminology as it is now is often outdated or kept too broad to be a good guidance for researchers and to successfully protect study participants. In a rapidly evolving field such as this, regular risk assessments and revision of laws based on correspondence with active researchers of the topic is inevitable. Clear and thorough laws against genome-based discrimination are particularly important since protecting an individual's privacy can, in practice, oftentimes not be fully assured, simply due to the fact that technologies and methods available to attackers in the future can only be speculated about. Therefore, it is even more important to instantiate laws that explicitly prevent discrimination in the case of data leakage, to provide a fail-safe system in cases where privacy protecting measures fall short (Figure 1f(Fig. 1)). First steps into this direction have already been made by means of the GDPR, ALTAI and AI Act as outlined above.

## Conclusion

Putting the risks of re-identification using genomic sequence data into perspective, data privacy is a concern that needs to be taken seriously. At this point in time, while all the above-mentioned methods are eligible threats, the costs involved and the expertise needed are still rather high and one could wonder, how realistic the threat really is. But as always in the field of data security and privacy, it is not only important to assess what *is* but also what potentially *will be*. While today, the motivation to acquire the genomic sequence of most people is considerably low given the entailed costs and effort, further decrease in sequencing price, increased insight into the genome and better computing performance are likely to make it more attractive in the future. The future in mind, present day scientists are required to address the issues of genomic data privacy to assure responsible research. This entails active communication with lawmakers to provide non-discrimination laws that protect study participants in the case of data leakage and which are up to date with the science. Further, increased collaboration of data security and privacy researchers with life scientists is essential to develop privacy-preserving data sharing techniques that are specifically tailored to genomics data in the near future. In this context, we can expect an increased necessity for bioinformaticians, computational biologists, biomathematicians, and others to optimally communicate the needs of the genomics community to computer scientists, in order to enable easier, yet secure sharing of genomic data. 

## Acknowledgements

This work was supported by the HGF Helmholtz AI grant Pro-Gene-Gen (ZT-I-PF-5-23), the HGF Incubator grant sparse2big (ZT-I-0007), HGF Incubator grant “Trustworthy Federated Data Analytics (TFDA)" (ZT-I-OO1 4), by NaFoUniMedCovid19 (FKZ: 01KX2021, project acronym COVIM), by the German Research Foundation (DFG) (INST 37/1049-1, INST 216/981-1, INST 257/605-1, INST 269/768-1, INST 217/988-1, INST 217/577-1, INST 217/1011-1, INST 217/1017-1 and INST 217/1029-1), ImmunoSep (grant 84722) and the BMBF-funded excellence project Diet-Body-Brain (DietBB) (grant 01EA1809A).

The figure was created with BioRender.com.

## Conflict of interest

The authors declare that they have no conflict of interest.
